# Immunohistochemical analysis of osteoclastic and osteoblastic activity in ossifying fibroma and juvenile ossifying fibroma: A comparative study

**DOI:** 10.25122/jml-2023-0126

**Published:** 2023-09

**Authors:** Jawaher Tater, Ameena Ryhan Diajil

**Affiliations:** 1 Department of Oral and Maxillofacial Pathology, College of Dentistry, University of Baghdad, Baghdad, Iraq; 2 Department of Oral Medicine, College of Dentistry, University of Baghdad, Baghdad, Iraq

**Keywords:** cemento-ossifying fibroma, juvenile ossifying fibroma, RANK, RANK ligand, osteoprotegerin, COF: Cemento-Ossifying Fibroma, JOF: Juvenile Ossifying Fibroma, PsJOF: Psammomatoid Juvenile Ossifying Fibroma, TrJOF: Trabecular Juvenile Ossifying Fibroma, RANK: Receptor Activator of Nuclear Factor-kB, RANKL: RANK Ligand, OPG: Osteoprotegerin

## Abstract

Cemento-ossifying fibroma (COF) and juvenile ossifying fibroma (JOF) have been considered distinct entities within the category of fibro-osseous lesions. This study aimed to assess osteoblast and osteoclast activity in COF and JOF by investigating bone resorption markers, specifically receptor activator of nuclear factor-kB (RANK), RANK ligand (RANKL), and its inhibitor osteoprotegerin (OPG). A comparative analysis of these markers was performed on all lesions. Immunohistochemistry was employed to evaluate and quantify the expression of these biomarkers in a sample of 20 cases of cemento-ossifying fibroma (COF), 15 cases of psammomatoid juvenile ossifying fibroma (PsJOF), and 10 cases of trabecular juvenile ossifying fibroma (TrJOF). The expression of osteoprotegerin was significantly higher in cemento-ossifying fibroma (33.9±13.0) compared to trabecular juvenile ossifying fibroma (27.3±9.2) and psammatoid ossifying fibroma (25.2±14.9), with the COF showing the highest expression followed by the latter two (p=0.037). There was a higher percentage (80%) of stromal fibroblast cells that showed positive expression of RANKL in cemento-ossifying fibroma (COF) compared to psammomatoid juvenile ossifying fibroma (PsJOF) (33.3%) and trabecular juvenile ossifying fibroma (TrJOF) (30.0%) when considering a positive expression score of 3 (p=0.024). Cemento-ossifying fibroma demonstrated the highest expression of osteoprotegerin and RANKL-positive stromal fibroblast cells, followed by psammomatoid juvenile ossifying fibroma and trabecular juvenile ossifying fibroma. These findings provide valuable insights into the pathogenesis of these lesions.

## INTRODUCTION

An ossifying fibroma is a tumor, typically well-defined or encapsulated in rare cases, composed of fibrous tissue and varying levels of mineralized material resembling bone, cement, or both [1]. Three clinicopathologic classifications are used to categorize ossifying fibromas. The term “cemento-ossifying” is still utilized in the most recent World Health Organization (WHO) classification (2022) to describe a variant that develops in the parts of the jaws supporting teeth and is believed to originate from odontogenic tissues. The term “juvenile” has been eliminated from the classification, given that psammomatoid variants can occur across a wide age range [2].

Psammomatoid juvenile ossifying fibroma (PsJOF) and trabecular juvenile ossifying fibroma (TrJOF) are two subtypes of juvenile aggressive ossifying fibroma (JAOF). Because JAOFs are aggressive but benign tumors, a clinicopathological correlation is necessary to make the correct diagnosis [3].

Research has highlighted the significance of the receptor-activated nuclear kappa B (RANK) and its ligand (RANKL) in promoting the growth and activation of osteoclasts. RANKL binds to RANK on the surface of preosteoclasts, facilitating osteoclast activation and development [4]. Emerging evidence suggests that RANK, RANK ligand, and osteoprotegerin (OPG) play a significant role in the etiology of oral diseases characterized by bone resorption [5]. A disruption in the quantity and activity of osteoclastic cells leads to incorrect bone resorption that exceeds the ability of osteoblasts to compensate in the majority of bone disorders [6, 7].

The activity of RANKL can be counteracted by the soluble decoy receptor Osteoprotegerin (OPG), which binds to RANKL, preventing it from interacting with RANK and thereby inhibiting the differentiation of osteoclasts [8].

There is a lack of studies exploring biomarkers in juvenile ossifying fibromas (JOF), and most research on biomarkers in ossifying fibromas has focused on the adult subtype. This is likely due to the rarity of JOF and the difficulty in obtaining tissue samples for analysis. Nevertheless, some research has suggested a potential role for the Activator of Nuclear Factor-kappa B Receptor Ligand (RANKL) signaling pathway in JOF biology. RANKL is a regulator of osteoclastogenesis, the process by which bone-resorbing cells called osteoclasts are formed and activated. JOF is known to cause significant bone resorption and destruction, so that RANKL may be involved in the pathogenesis of these tumors. Further research is needed to better understand the role of biomarkers in JOF and identify potential therapeutic targets [9, 10].

Bone development and turnover are tightly controlled by the receptor-activator of the nuclear kappa B ligand (RANKL) pathway. By stimulating the differentiation of mononuclear preosteoclasts and macrophages into multinucleated osteoclasts, RANKL plays a crucial role in the osteoclastogenesis process. In pathological bone diseases, this may cause bone resorption and destruction. The soluble tumor necrosis factor receptor superfamily member osteoprotegerin (OPG) mimics the actions of RANKL by blocking its binding to its target cells. Both OPG and RANKL are inhibitors of osteoclast differentiation [11].

## MATERIAL AND METHODS

### Samples

The samples for this retrospective study were provided by the Oral Pathology Laboratories of the College of Dentistry, University of Baghdad, and Al-Shaheed Ghazi Hospital for Specialty Operations. These samples were collected between December 2018 and December 2020. It is crucial to emphasize that all study participants were not exposed to medical or pharmaceutical interventions.

The study included a total of 45 samples, comprising 20 cases of cemento-ossifying fibroma (COF), 10 cases of trabecular juvenile ossifying fibroma (TrJOF), and 15 cases of psammomatoid juvenile ossifying fibroma (PsJOF). An independent examiner reviewed the slides stained with hematoxylin and eosin to confirm the diagnosis. The samples, prepared as 5-micrometer sections, were analyzed and classified according to the WHO classification [2].

### Immunohistochemistry

A detection system (Elabscience) was employed for immunohistochemistry, utilizing a mouse and rabbit-specific HRP/DAB detection IHC kit (E-IR-R220). This kit is designed to work seamlessly with primary antibodies from both rabbit and mouse sources.

The slides were treated with SP reagent B (peroxidase blocking buffer) at room temperature for 15 minutes to neutralize endogenous peroxidase activity. The primary antibody was then applied with the appropriate dilution ratio using SP reagent G (antibody dilution buffer). These primary antibodies included TNFRSF11A polyclonal antibody (RANK) diluted at 1:100, anti-RANKL (elabscience) diluted at 1:200, and TNFRSF11B polyclonal antibody (OPG) (Elabscience) diluted at 1:100.

Next, the slides were incubated at room temperature or 37°C for 30 minutes to 1 hour or at 4°C overnight (followed by a 30-minute re-warming step at 37°C). A drop of SP reagent C (poly peroxidase-anti-rabbit/mouse IgG) was applied, and the slides were incubated again at room temperature or 37°C for 30 minutes. To create the DAB working solution, one drop (approximately 50 µL) of SP reagent D (high-sensitive DAB concentrate) was added to each 1 mL of SP reagent E (high-sensitive DAB substrate). The components were thoroughly mixed, and the resulting tan or brownish-yellow solution indicated a positive signal.

After washing the section with distilled water (DI), it was treated with SP reagent F (hematoxylin staining buffer) at room temperature for 5 to 10 minutes. Subsequently, the slides were rinsed with DI water. The slides were then placed in alkaline water for 1 minute and thoroughly washed with water for 5 to 10 minutes. Following this, a gradient alcohol dehydration process was carried out. A drop of neutral balsam was applied next to the tissue, and the slide was covered with a covered glass and left to dry.

### The staining analysis

The evaluation considered the count of positive and negative cells in the COF, PsJOF, and TrJOF samples. Quantitative analyses were performed on stromal fibroblast-like cells and bone matrix (cells around bone/osteoid-osteoblast and osteoclast). For stromal fibroblast-like cells, the proportion of stained cells was categorized as follows: 0, indicating no stained cells; 1, representing 25% stained cells; 2, denoting 25%-50% stained cells; or 3, indicating 50% stained cells. This evaluation method followed the approach outlined by Tobón-Arroyave *et al*. [12]. In the bone matrix, the analysis was expressed as the mean percentage of positive cells for RANK, RANKL, and OPG.

The evaluation of osteoblasts and osteoclast cells was expressed as the mean percentage of positive cells for RANK, RANKL, OPG, and OC. Specifically, five representative fields exhibiting the highest immunopositivity, encompassing regions from the periphery to demonstrate active bone resorption and formation, were selected and visualized with an integrated graticule measuring 0.0961 mm^2^ at 40x magnification. The percentage of positive cells was the ratio of the number of positively stained cells divided by the total number of cells counted (positive and negative osteoclast and osteoblast cells) and expressed as mean±standard deviation (SD).

### Statistical analysis

Statistical Package for Social Sciences (SPSS) was used to conduct the statistical analysis (version 20.0 for Windows, SPSS, Chicago, IL, USA). The distribution of osteoblasts, osteoclasts, and fibroblasts was assessed using the Kolmogorov-Smirnov test and found to be non-normal. Quantitative data were expressed as mean, standard deviation, and range for normally distributed data, while mean, standard deviation, and mean rank were used for non-normally distributed data. Differences between groups were analyzed using the Kruskal-Wallis test. Qualitative data were presented as counts and percentages and analyzed using the Chi-square test. A statistically significant result was defined as a p value of <0.05.

## RESULTS

### Clinical findings

The clinical characteristics are outlined in Table 1. There was a higher prevalence of female patients with COF, accounting for 70% (14/20), in contrast to male patients, which constituted 30% (6/20). Conversely, male patients were more predominant in PsJOF (60%, 9/15) and TrJOF (70%, 7/10). The mean age of patients with COF, TrJOF, and PsJOF was 27, 20, and 15 years, respectively.

**Table 1:** Clinical results of the investigated cases

		Type of ossifying fibroma					
		COF (n=20)		PsJOF (n=15)		TrJOF (n=10)	
		Count	%	Count	%	Count	%
Gender	Female	14	70.0%	6	40.0%	3	30.0%
	Male	6	30.0%	9	60.0%	7	70.0%
	Ratio	2:1		2:3		1:2	
Age		Mean (range)	SD	Mean (range)	SD	Mean (range)	SD
		27 (14-48)	10	20 (8-50)	11	15 (13-22)	3
Location	Mandible	13	65.0%	4	26.7%	6	60.0%
	Maxilla	7	35.0%	11	73.3%	4	40.0%

**Figure 1:**
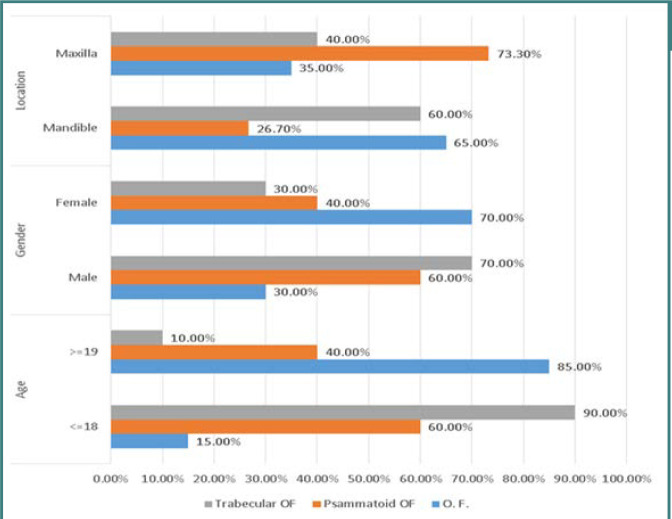
Demography and clinical location of ossifying fibroma and its subtypes

Regarding the location of the lesions, a significant proportion of COF (65%) and TrJOF (60%) cases were observed in the mandible, whereas the majority of PsJOF cases (73.3%) were identified in the maxilla (Figure 1).

**Figure 2:**
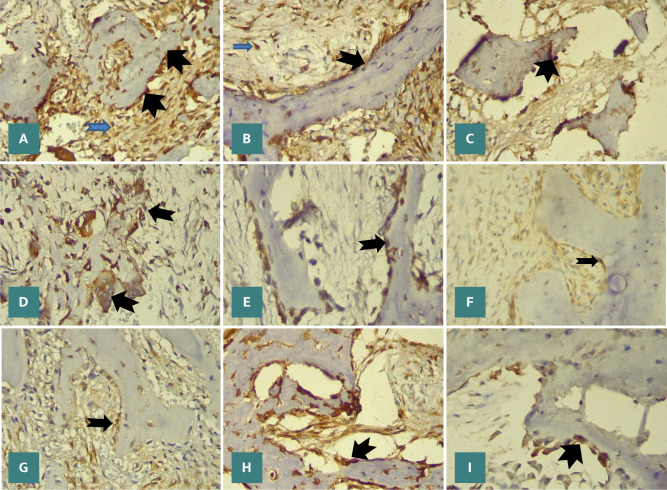
Immunohistochemical staining for RANK, RANKL, and OPG, respectively, in COF (A,B,C), TrJOF (D,E,F), and PsJOF (G,H,I). x40. Black arrowheads: positive cells around bone matrix; blue arrowheads: stromal fibroblast-like cells.

Qualitative and quantitative analyses were performed on osteoclasts, osteoblasts, and stromal fibroblast-like cells in the tissue samples. Immunohistochemical staining revealed the presence of resorption markers RANK, RANKL, and OPG in the cytoplasm and plasma membranes of stromal fibroblast-like cells, osteoclasts, and the bone surrounding osteoblasts within COF, TrJOF, and PsJOF tissue samples (Figure 2A-I).

When comparing the number of positive cells among the lesions using the Kruskal-Wallis test, a significant difference in OPG expression was observed (p<0.05). Specifically, the expression of OPG was higher in COF, followed by TrJOF and then PsJOF. Although not reaching statistical significance, RANK expression was higher in PsJOF, followed by TrJOF, and lower in COF samples. Similarly, RANKL expression was higher in TrJOF, followed by COF, and lower in PsJOF samples (Table 2).

**Table 2:** Comparative analysis of RANK, RANKL, and OPG immunoexpression in positive cells around bone trabecula for all lesions investigated

Markers	Type						p value
	COF		PsJOF		TrJOF		
	Mean±SD		Mean±SD		Mean±SD		
OPG	33.9 (28.2)	13.0	25.2 (16.7)	14.9	27.3 (22.1)	9.2	0.037
RANKL	62.2 (24.9)	11.3	53.2 (16.8)	17.1	64.2 (26.6)	7.4	0.103
RANK	70.2 (20.9)	14.9	74.7 (25.3)	11.3	73.2 (21.4)	10.5	0.580

Data expressed as mean±SD (mean rank)

The immunoreactivity of these markers in stromal fibroblast-like cells was examined, and the data were presented as both counts and percentages. A comparative analysis of the immunoexpression of these markers was carried out for all lesions in stromal fibroblast-like cells using the Chi-Square test (Table 3). There was a higher percentage (80%) of stromal fibroblast cells that showed positive expression of RANKL in COF compared to psammomatoid juvenile ossifying fibroma (PsJOF) (33.3%) and trabecular juvenile ossifying fibroma (TrJOF) (30.0%) when the positive expression rate was scored as 3.

**Table 3:** Comparative analysis of RANK, RANKL, and OPG immunoexpression in stromal fibroblast-like cells across all lesions

Fibroblast score		Type						p value
		COF		PsJOF		TrJOF		
		Count	%	Count	%	Count	%	
OPG	0	9	45.0%	9	60.0%	7	70.0%	0.393
	1	9	45.0%	4	26.7%	1	10.0%	
	2	2	10.0%	2	13.3%	2	20.0%	
	3	0	0.0%	0	0.0%	0	0.0%	
RANKL	0	0	0.0%	1	6.7%	0	0.0%	0.024
	1	2	10.0%	4	26.7%	1	10.0%	
	2	2	10.0%	5	33.3%	6	60.0%	
	3	16	80.0%	5	33.3%	3	30.0%	
RANK	0	0	0.0%	1	6.7%	3	30.0%	0.101
	1	1	5.0%	3	20.0%	2	20.0%	
	2	5	25.0%	3	20.0%	1	10.0%	
	3	14	70.0%	8	53.3%	4	40.0%	

Data expressed as numbers and percentages

## DISCUSSION

According to the current study, patients diagnosed with COF had a mean age of 27 years, while those with juvenile forms of OF, such as PsJOF and TrJOF, had mean ages of 20 and 15 years, respectively. These findings are consistent with previous studies, which reported that TrJOF is typically seen in children and adolescents, while PsJOF affects a wider range of ages [13-15]. The latest WHO classification, as mentioned by Chi *et al*., has renamed PsJOF as PsOF, reflecting the broader age range affected by this type of fibroma [16].

The current study assessed RANK-RANKL-OPG in cemento-ossifying fibroma and juvenile ossifying fibroma. In terms of the bone matrix, OPG demonstrated significant differences among these lesions, with notably higher expression in COF samples, followed by TrJOF and then PsJOF. This finding confirms that juvenile ossifying fibromas tend to exhibit a more osteolytic nature, as the presence of OPG molecules can inhibit osteolytic lesions. Most of the time increased RANKL and decreased OPG levels lead to bone loss [17]. Conversely, when these lesions were compared, the differences in RANKL and RANK immunoexpression were not significant. However, RANKL immunoexpression was higher in TrJOF, while RANK was more pronounced in PsJOF. This data may indicate an increase in bone resorptive activity in juvenile ossifying fibroma. This result is consistent with the study conducted by Elias *et al*. [18], where they found that the expression of RANK played a pivotal role in osteolytic activity. This finding helps elucidate the aggressive behavior, high rate of bone resorption, and recurrence observed in these lesions.

High RANK expression in juvenile ossifying fibroma suggests denosumab as a potential treatment, as it can inhibit osteoclast activity. However, decisions regarding its use should be guided by healthcare professionals and consider individual patient factors. While denosumab holds promise, further research is crucial to evaluate its efficacy and safety in both conditions [19].

In this study, stromal fibroblast-like cells in bone lesions were assessed. The positive immunostaining of RANK, RANKL, and OPG in these fibroblast-like cells was compared among COF, TrJOF, and PsJOF. Our findings revealed a significantly higher expression of RANKL in COF compared to PsJOF and TrJOF. *In vivo* experiments by Hofbauer and Baud’huin *et al*. [20, 21] suggested that RANKL could act as a pro-resorption factor. According to their findings, higher RANKL expression might be linked to enhanced osteoclast activity, potentially increasing an individual's susceptibility to resorption, which is supported by a previous study by Bartolini *et al*. [22], who suggested that OF originates from multipotent mesenchymal cells of periodontal origin, capable of forming both bone and cementum. This hypothesis is supported by the work of Mintz and Velez [23]. Anubha *et al*. [24] provided further elaboration, stating that the fibrous connective tissue of the periodontal membrane primarily consists of collagen, oxytalan, mucopolysaccharides, and cells capable of producing bone, cement, and fibrous tissue. Under pathological circumstances, such blast cells can produce tumors made of fibrous tissue, cementum, and lamellar bone. Additionally, the study found a higher percentage of stromal fibroblast cells showing positive expression of RANKL in cemento-ossifying fibroma compared to psammatoid juvenile ossifying fibroma and trabecular juvenile ossifying fibroma. RANKL is a protein that stimulates the activity of osteoclasts, which are also involved in bone resorption. This finding may suggest that ossifying fibroma lesions have a higher potential for bone resorption compared to the other two types of lesions.

The statistically significant difference in stromal fibroblast cell expression among the different types of lesions, as indicated by the Chi-square test, reinforces the idea that these lesions have unique molecular profiles that distinguish them. Overall, these findings contribute to our understanding of the molecular mechanisms underlying the development and progression of ossifying fibroma lesions, which can potentially inform the development of targeted therapies for this condition.

## CONCLUSION

The study results suggest that there is a balance between RANKL and OPG expression that regulates bone homeostasis. When RANKL is upregulated, and OPG is downregulated, it can lead to bone loss. Therefore, the findings of this study may provide insight into the molecular mechanisms underlying the pathogenesis of these lesions and may have implications for the development of novel treatments for these conditions. Further research is needed to understand better the complex interactions between RANK-RANKL-OPG and bone homeostasis in these lesions.
